# Geometric Determinants of *In-Situ* Direct Laser Writing

**DOI:** 10.1038/s41598-018-36727-z

**Published:** 2019-01-23

**Authors:** Andrew C. Lamont, Abdullah T. Alsharhan, Ryan D. Sochol

**Affiliations:** 1 Department of Mechanical Engineering, 2147 Glenn L. Martin Hall, University of Maryland, College Park, MD 20740 USA; 2 Fischell Department of Bioengineering, 2147 Glenn L. Martin Hall, University of Maryland, College Park, MD 20740 USA

## Abstract

Direct laser writing (DLW) is a three-dimensional (3D) manufacturing technology that offers significant geometric versatility at submicron length scales. Although these characteristics hold promise for fields including organ modeling and microfluidic processing, difficulties associated with facilitating the macro-to-micro interfaces required for fluid delivery have limited the utility of DLW for such applications. To overcome this issue, here we report an *in-situ* DLW (*is*DLW) strategy for creating 3D nanostructured features directly inside of—and notably, fully sealed to—sol-gel-coated elastomeric microchannels. In particular, we investigate the role of microchannel geometry (*e.g*., cross-sectional shape and size) in the sealing performance of *is*DLW-printed structures. Experiments revealed that increasing the outward tapering of microchannel sidewalls improved fluidic sealing integrity for channel heights ranging from 10 *μ*m to 100 *μ*m, which suggests that conventional microchannel fabrication approaches are poorly suited for *is*DLW. As a demonstrative example, we employed *is*DLW to 3D print a microfluidic helical coil spring diode and observed improved flow rectification performance at higher pressures—an indication of effective structure-to-channel sealing. We envision that the ability to readily integrate 3D nanostructured fluidic motifs with the entire luminal surface of elastomeric channels will open new avenues for emerging applications in areas such as soft microrobotics and biofluidic microsystems.

## Introduction

Recent advances in the capabilities of additive manufacturing or “three-dimensional (3D) printing” technologies have dramatically expanded the degree of architectural freedom with which researchers can design and manufacture systems at micron-to-submicron scales^1,2^. At present, extrusion-based methods (*e.g*., direct ink writing) have garnered significant interest in the scientific community due to the vast material selection and low equipment costs afforded by such approaches^3–5^. The key limitations, however, stem from the condition that the nozzle be physically positioned at each location of material deposition, which not only increases printing times, but also prevents the fabrication of structures for which nozzle access is obstructed^6,7^. In addition, challenges associated with nozzle-material interactions and controls have typically restricted the utility of extrusion-based methods to structures with feature sizes of approximately 10 *μ*m or larger^8^. Thus, for 3D printing applications at smaller scales, researchers have focused on utilizing an alternative technology: direct laser writing (DLW)^9^.

DLW is a 3D manufacturing approach that relies on using tightly focused femtosecond laser pulses to initiate spatially controlled polymerization of a liquid-phase photocurable material *via* two-photon (or multi-photon) absorption phenomena^10^. By precisely positioning the laser focal point at designated locations, 3D structures comprised of cured material can ultimately be produced with feature resolutions down to the sub-100 nm range^11^. For micron- and submicron-scale fluidic applications, however, this resolution results in an inherent trade-off that limits or prevents the incorporation of the macro-to-micro interfaces that are critical for delivering fluid volumes (*e.g*., chemicals, reagents, wash buffers, particle suspensions, *etc*.) into enclosed DLW-manufactured systems^12,13^. Consequently, DLW-based studies have predominantly involved the use of unenclosed micro- and nanostructures^14–16^. Nonetheless, due to the potential of DLW for fluidic applications^17^, an increasing number of groups have developed methods to facilitate the aforementioned macro-to-micro interfaces.

Previously, researchers have reported two main classes of techniques for using DLW to additively manufacture fluidic systems: (*i*) full device printing, and (*ii*) *in-situ* fabrication. Recently, Marino *et al*. demonstrated an example of the former in which a complete system comprised of 3D microfluidic blood-brain barrier models that are fully integrated with larger-scale coupling ports (*i.e*., for the manual insertion of external tubing) was constructed in a single print run^18^. Although only two coupling ports were manufactured (one input and one output), using DLW to generate multiple structures with feature sizes in the meso-to-milliscale range typically requires print times on the order of days–times that would be compounded in cases that demand additional input and/or output coupling ports. As a result, researchers have primarily refrained from using DLW exclusively to manufacture entire devices, opting instead for *in-situ* fabrication schemes^19,20^.

*In-situ* DLW (*is*DLW) encompasses a variety of approaches that involve first manufacturing a microfluidic channel using alternative fabrication processes (*e.g*., micromolding or laser ablation), then inputting a photocurable material into the microfluidic channel, and lastly, using DLW to print structures directly inside of the channel^21^. For example, researchers have employed soft lithography protocols with poly(dimethylsiloxane) (PDMS) to demonstrate *is*DLW with PDMS-on-glass microchips^22,23^. One challenge associated with the gas permeability of PDMS is that a thin oxygen layer on the channel surface can disrupt photopolymerization phenomena, which while beneficial to applications including optofluidic lithography and continuous liquid interface production^24–26^, can lead to print failures for *is*DLW. Consequently, *is*DLW for PDMS-on-glass systems typically involves printing structures onto the glass surface (rather than PDMS surfaces) of the microchannel interior^22,23^. Other groups have developed PDMS-photoresist-glass sandwich-chip approaches in which 3D structures are first printed in unenclosed photoresist-on-glass channels, and then a PDMS slab is sealed atop the photoresist to form enclosed microchannels (with DLW-based structures sealed only to the photoresist sidewalls and glass base)^27–29^. Alternatively, fully glass microchips can be used for *is*DLW^30–33^; however, the methods for manufacturing glass microdevices (*e.g*., wet etching and laser ablation) can be exceedingly time, labor, and cost-intensive, while necessitating access to advanced fabrication facilities^34^.

The efficacy of *is*DLW for fluidic applications is predicated on new methods that yield full sealing interactions between DLW-printed structures and the entire luminal surface of the microchannel, while bypassing limitations inherent to glass microchip fabrication. Recently, we observed that using *is*DLW to build microfluidic structures within soft lithography-based PDMS-on-glass devices can lead to malformed prints at taller heights (≥50 *μ*m), and consistent with prior works, diminish sealing performance at PDMS interfaces^35^. To limit the effects of these failure modes, Lölsberg *et al*. utilized a PDMS-on-glass device with trapezoidal microchannels (~30 *μ*m in height) to manufacture a microfluidic spinneret head *via* an inverted *is*DLW process; however, persisting sealing issues necessitated the inclusion of additional intersecting sacrificial channels for silane-based glues to be manually loaded in an effort to improve mechanical and sealing integrity^36^. Although fluidic experimentation (*e.g*., burst-pressure measurements) to assess the influence of the silane-based glue on sealing performance was not reported, the results suggest a possible role for microchannel geometry in *is*DLW.

**Figure 1:**
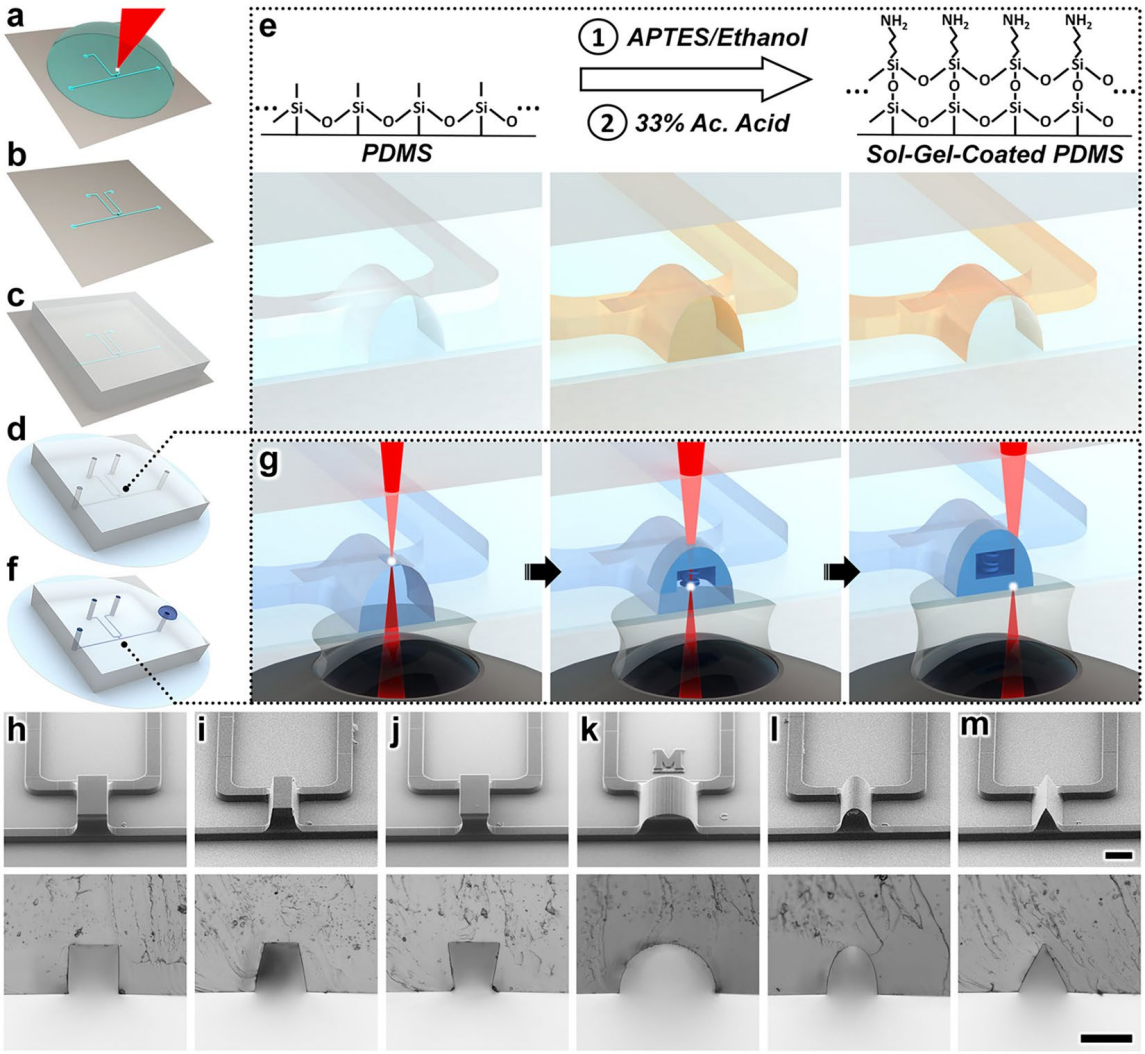
Sol-gel-based *in-situ* direct laser writing (*is*DLW) concept. (**a**–**g**) Illustrations of the *is*DLW fabrication protocol for a microfluidic element printed inside a semi-ovular microchannel. (**a**) DLW of the channel mold structures. (**b**) Fabricated negative master mold. (**c**) Micromolding of poly(dimethylsiloxane) (PDMS). (**d**) Micromolded PDMS bonded to a glass substrate. (**e**) Acetic (Ac.) Acid-catalyzed sol-gel reaction for coating the PDMS microchannels with an adhesive layer of (3-Aminopropyl)triethoxysilane (APTES). (**f**) Vacuum loading of a liquid-phase photocurable material into the sol-gel-coated microchannels. (**g**) The “ceiling-to-floor” *is*DLW process. Focused femtosecond laser pulses (*red*) pass through an objective lens, immersion oil, glass substrate, and liquid-phase photomaterial to initiate spatially controlled photopolymerization (*white*) in a point-by-point, layer-by-layer methodology, ultimately producing a structure comprised of cured photomaterial (*blue*) that is fully sealed to the entire luminal surface of the sol-gel-coated microchannel. (**h**–**m**) Micrographs of (*Top*) DLW-printed negative master molds, and (*Bottom*) replicated PDMS profiles corresponding to distinct microchannel cross-sectional geometries: (**h**) rectangular (deep reactive-ion etching (DRIE) mimetic), (**i**) outward-tapered (positive-tone photoresist mimetic), (**j**) inward-tapered (negative-tone photoresist mimetic), (**k**) semi-circular, (**l**) semi-ovular, and (**m**) triangular (*see also* Supplementary Fig. S1). Scale bars = 100 *μ*m.

In this work, we present a novel sol-gel-based *is*DLW strategy that enables 3D nanostructured designs to be printed inside of, and fully sealed to, PDMS-on-glass microchannels (Fig. 1a–g). We utilize this approach to investigate the effects of microchannel geometric factors–namely, channel height and cross-sectional shape–on the sealing performance of *is*DLW-printed structures. Specifically, we employ theoretical and experimental methods to characterize the microfluidic sealing integrity of *is*DLW-printed 10-*μ*m-thick barrier wall structures sealed to sol-gel-coated PDMS-on-glass microchannels with heights of 10, 25, 50, and 100 *μ*m, and six distinct cross-sectional profiles. Three of the microchannel profiles are inspired by those resulting from conventional soft lithography protocols corresponding to: (*i*) deep reactive-ion etching (DRIE) processes that produce relatively straight channel sidewalls^37^ (Fig. 1h), (*ii*) positive-tone photoresists that result in sidewalls that are slightly tapered outward^38^ (Fig. 1i), and (*iii*) negative-tone photoresists that result in inward-tapered sidewalls^39^ (Fig. 1j). Additional microchannel profiles that feature outward-tapering geometries include semi-circular (Fig. 1k), semi-ovular (Fig. 1l), and triangular (Fig. 1m) cross-sections. Lastly, as an exemplar, we examine the flow rectification performance of an *is*DLW-printed 3D microfluidic coil spring diode for which functionality is inextricably linked to luminal microchannel sealing. The presented sol-gel-based *is*DLW protocols and results provide a critical foundation for researchers to bypass the challenges associated with achieving micro-to-macro interfaces and microfluidic sealing, and ultimately, leverage the 3D geometric and scaling-induced benefits of DLW for a diversity of chemical, mechanical, and biological fluidic applications.

## Results

### Sol-gel-based *in-situ* direct laser writing (*is*DLW)

The methodology presented here utilizes two distinct DLW approaches for two different fabrication roles: (*i*) Dip-in Laser Lithography (DiLL) mode-based DLW for channel mold manufacturing, and (*ii*) *is*DLW for microstructure 3D printing. Previously, several research groups have demonstrated the use of DLW for generating microchannel master molds with arbitrary geometries for elastomer replication^36,40–43^. In this work, we utilized the negative-tone photoresist, IP-S, to print negative master molds onto Si substrates *via* DiLL mode-based DLW (Fig. 1a). Following development (Fig. 1b), the negative master was used to micromold the silicone elastomer, PDMS (Fig. 1c). After curing, the PDMS was removed from the molds, hole-punched at inlet and outlet locations, and then plasma-bonded to borosilicate glass substrates (Fig. 1d). In prior reports, researchers have presented a variety of protocols for applying sol-gel coatings to microfluidic channels^44–46^. In this study, we employed an acid-catalyzed sol-gel reaction developed by Beal *et al*.^47^ to chemically coat the inner surface of the PDMS microchannel with (3-Aminopropyl)triethoxysilane (APTES) (Fig. 1e). Thereafter, we applied previously described microfluidic vacuum-loading approaches^48^ to infuse the liquid-phase photoresist, IP-L 780, into the microchannels (Fig. 1f). For the oil-immersion mode-based *is*DLW step, we utilized a “ceiling-to-floor” DLW strategy in which structures were printed starting at the tallest point of the sol-gel-coated PDMS microchannel (Fig. 1g–*left*). The point-by-point, layer-by-layer DLW routine followed a layering sequence from the top down, with attachment to the glass substrate occurring at the end of the printing process (Fig. 1g–*right*). After development to remove any residual uncured photoresist, the devices were ready for use and did not require any additional post-processing (*e.g*., with sealant glues)^36^.

**Figure 2:**
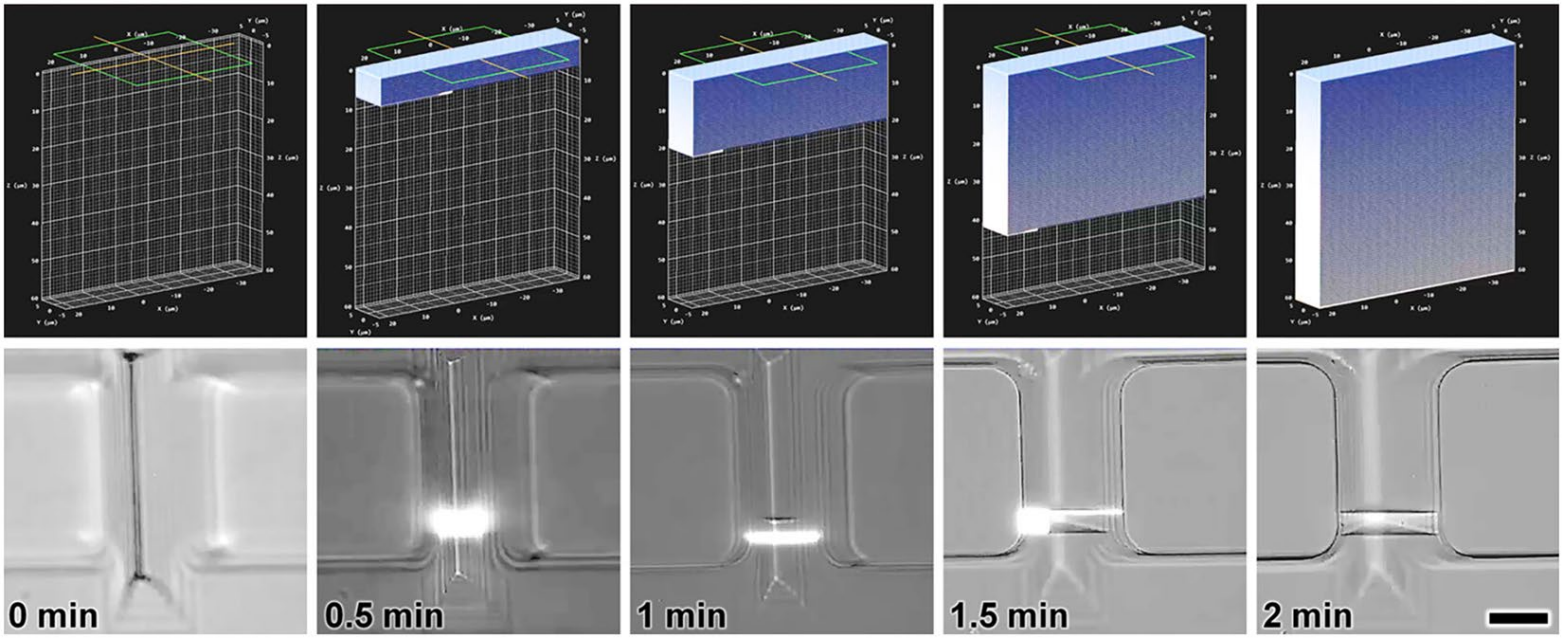
Sequential computer-aided manufacturing (CAM) simulations (*Top*) and corresponding *is*DLW fabrication results (*Bottom*) for printing a microfluidic barrier wall structure (10 *μ*m in thickness) within a sol-gel-coated PDMS microchannel with a triangular cross-section and height of 50 *μ*m (*see also* Supplementary Movie S1). Scale bar = 25 *μ*m.

Representative fabrication results for DLW-printed negative master molds and corresponding replicated PDMS for the six microchannel profiles examined in this study are presented in Fig. 1h–m *top* and *bottom*, respectively. Additional results for replicated PDMS cross-sections corresponding to every additional microchannel height and geometry investigated are included in Supplementary Fig. S1. The efficacy of the “ceiling-to-floor” *is*DLW protocol is directly linked to the adhesion between the cured photoresist and the top of the PDMS channel, which serves as an anchoring substrate during the layer-by-layer printing process (Fig. 1g). To initially characterize the influence of the sol-gel coating on the adhesion dynamics, we performed *is*DLW test prints using both uncoated and sol-gel-coated PDMS microchannels. The *is*DLW fabrication results for the uncoated PDMS-on-glass devices revealed structure detachment from the top surface of the microchannel during the printing process–a critical failure mode (Supplementary Fig. S2). In contrast, we did not observe such print failures for cases in which the PDMS microchannels included the sol-gel coating (*e.g*., Fig. 2).

### Sealing efficacy *versus* microchannel geometry

To elucidate the role of microchannel geometry in the fluidic sealing performance of *is*DLW-printed structures, we performed burst-pressure experiments for 10-*μ*m-thick fluidic barrier walls constructed in sol-gel-coated PDMS-on-glass microchannels with varying height and cross-sectional shape. We utilized computer-aided manufacturing (CAM) methods to convert 3D models of the barriers to the writing-path code that governs the point-by-point, layer-by-layer positioning of the laser during *is*DLW fabrication. Four primary sets of code were generated corresponding to the four microchannel heights tested: 10, 25, 50, and 100 *μ*m. Specifically, to maintain consistency among experiments, identical barrier wall writing-path code was used for each cross-sectional profile of a given height, with the exception of the semi-circular profiles, which necessitated unique writing-path codes to account for the significantly larger channel widths. The writing-path code was designed for rectangular barrier structures that are slightly larger in width and height than the microchannel cross-sections (Fig. 2–*top*), which resulted in the laser focal point being positioned at various locations inside of the solid PDMS throughout the printing process. Because the photoresist was not present at such locations, however, barrier wall fabrication was inherently restricted to the photoresist-filled microchannel interior, thereby ensuring that the resulting barrier geometry conformed to that of the local channel profile (Fig. 2–*bottom*; Supplementary Movie S1).

**Figure 3:**
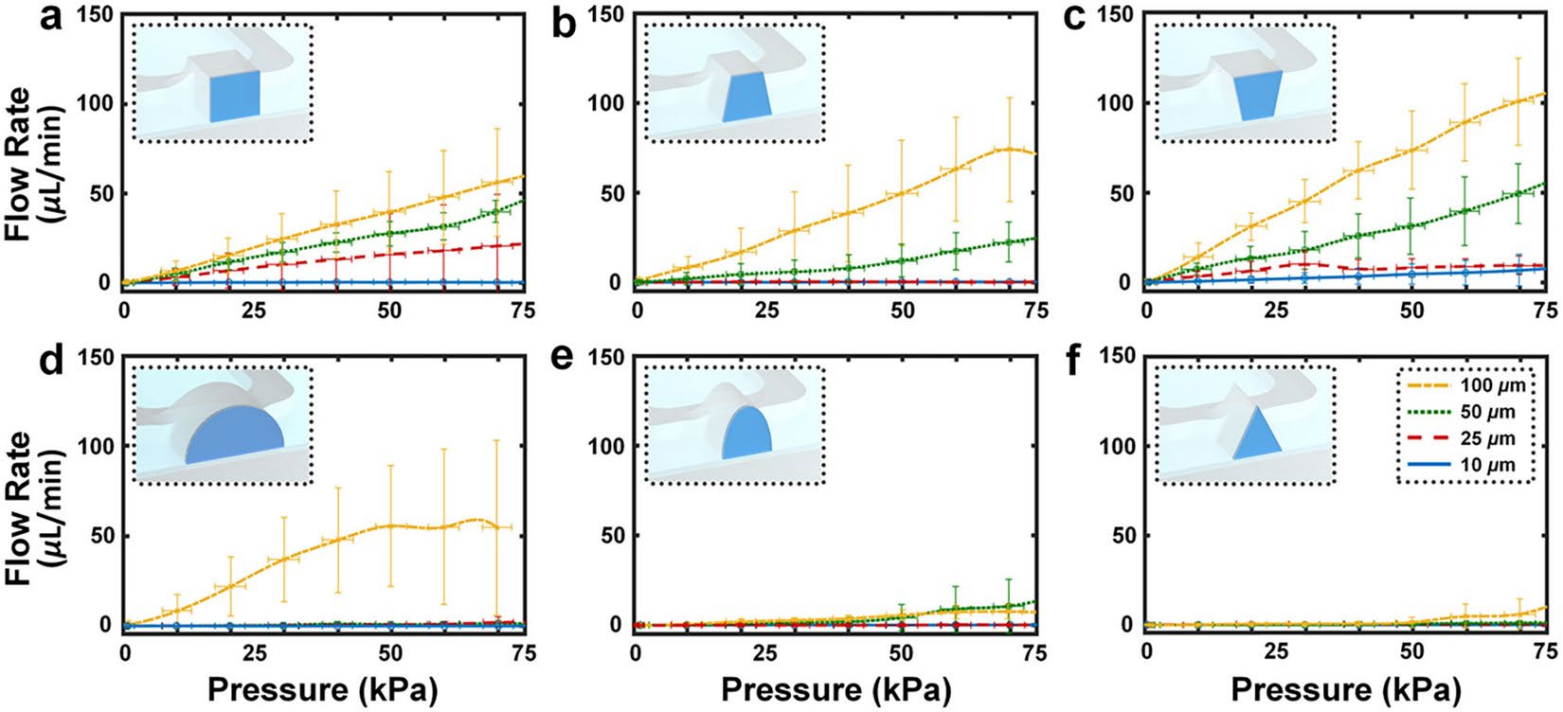
Experimental results for burst-pressure quantification of *is*DLW-printed microfluidic barrier wall structures (10 *μ*m in thickness) corresponding to sol-gel-coated PDMS microchannels with 10 *μ*m (*blue*), 25 *μ*m (*red*), 50 *μ*m (*green*), and 100 *μ*m (*yellow*) channel heights, and (**a**) rectangular, (**b**) outward-tapered, (**c**) inward-tapered, (**d**) semi-circular, (**e**) semi-ovular, and (**f**) triangular cross-sectional profiles. (*Insets*) Conceptual illustrations of the microfluidic barrier wall structures (*dark blue*) and microchannel cross-sections corresponding to each profile. Scale bars denote standard deviation corresponding to experiments with three different devices.

To quantify the burst-pressure dynamics associated with each microchannel height and cross-sectional profile, we incrementally increased the input pressure from 0 kPa to 75 kPa on one side of the *is*DLW-printed barrier wall, while simultaneously monitoring the rate of fluid flow passing (*i.e*., leaking) through the barrier structure (Fig. 3). The experimental results revealed three general trends. First, for cases in which fluid leaked past the barrier, we did not observe the types of abrupt changes in flow rate (*e.g*., a dramatic increase in fluid flow at a critical pressure due to full detachment and dislodgement of the barrier) that are characteristic of fluidic burst-pressure experiments^49^. Instead, we found that the magnitude of leakage flow gradually increased with rising input pressure in such cases. Secondly, decreasing the height of a microchannel for a given profile yielded improvements in the sealing integrity of the 10-*μ*m-thick barrier structures. Lastly, increasing the outward tapering of the microchannel sidewalls typically resulted in enhanced sealing performance (Fig. 3).

The three conventional microfabrication-inspired channel profiles provided preliminary insight into the sidewall tapering effects (Fig. 3a–c). For example, we observed that the overall sealing dynamics for the rectangular (DRIE mimetic) channel profile (Fig. 3a) appeared to be inferior to those of the outward-tapered (positive-tone photoresist mimetic) profile (Fig. 3b), while slightly superior to those of the inward-tapered (negative-tone photoresist mimetic) profile (Fig. 3c). This trend continued for the semi-circular (Fig. 3d), semi-ovular (Fig. 3e), and triangular (Fig. 3f) cross-sectional profiles. For channel heights up to 50 *μ*m, the barrier wall in the semi-circular microchannel effectively obstructed fluid flow for the pressures tested (Fig. 3d). Due to the 200 *μ*m width of the 100-*μ*m-tall semi-circular channel, a stitching approach was needed to print the barrier structure in two parts–the only case in this study for which a complete barrier could not be printed in a single step (Supplementary Movie S2). Burst-pressure testing revealed leakage flow from the onset of input pressure (Fig. 3d–*yellow*). Both the semi-ovular (Fig. 3e) and triangular (Fig. 3f) channel profiles did not exhibit significant changes in sealing performance over the range of microchannel heights investigated, demonstrating improved sealing efficacy compared to the microchannel profiles inspired by conventional microfabrication techniques (Fig. 3).

### Flow rectification dynamics for an *is*DLW-based 3D microfluidic coil spring diode

A number of groups have demonstrated the considerable advantages associated with using additive manufacturing technologies for the fabrication and integration of microfluidic circuit elements^50–53^. Previously, we introduced the first 3D printed microfluidic diode–a fluidic circuit element that passively allows fluid to flow in one direction, while obstructing flow in the opposite direction^54^. Despite the functionalities enabled by prior 3D printed microfluidic circuits, the size of such systems has remained relatively large in the meso- to millimeter-scale range. To explore the potential of leveraging *is*DLW to drastically reduce the size of 3D microfluidic circuity, we designed and printed 3D microfluidic coil spring diodes inside of sol-gel-coated PDMS-on-glass microchannels.

**Figure 4:**
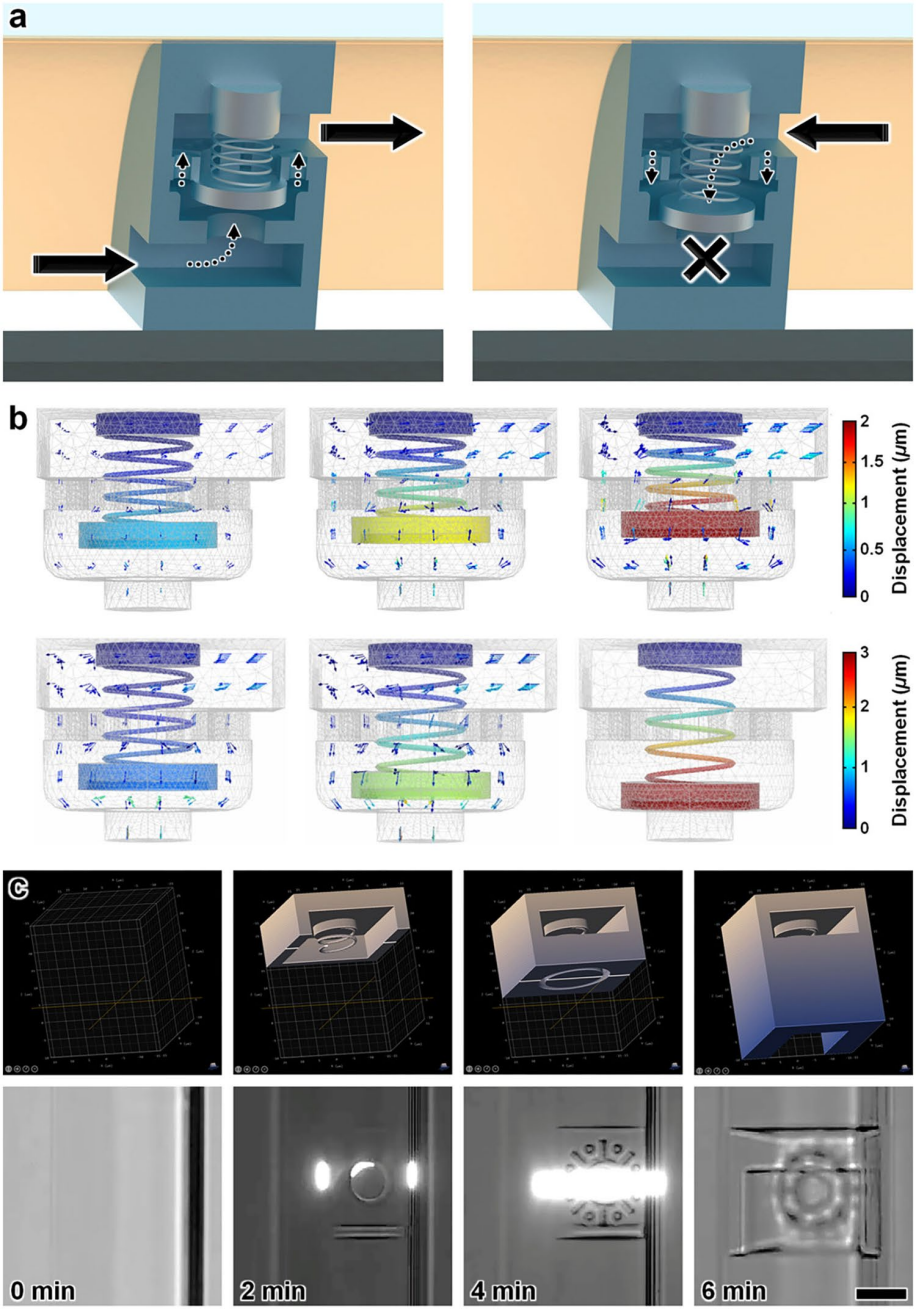
*Is*DLW-based 3D microfluidic coil spring diode. (**a**) Conceptual illustrations of flow rectification functionality. (*Left*) Under forward flow conditions, the helical coil spring compresses as the sealing disc is directed away from the bottom orifice, thereby permitting fluid flow through the radially arrayed through-holes and then out the top orifice. (*Right*) Under reverse flow conditions, the helical coil spring expands as the blocking disc forms a fluidic seal at the bottom orifice, which physically obstructs the flow of fluid through the element. (**b**) Sequential COMSOL Multiphysics fluid-structure interaction (FSI) simulations of flow dynamics corresponding to forward flow (*Top*) and reverse flow (*Bottom*) (*see also* Supplementary Movie S4). Arrows denote fluid velocity field vectors. (**c**) Sequential CAM simulations (*Top*) and corresponding *is*DLW fabrication results (*Bottom*) for printing a 3D microfluidic coil spring diode within a sol-gel-coated PDMS microchannel with a semi-ovular cross-sectional profile and height of ~25 *μ*m (*see also* Supplementary Movie S5). Scale bar = 10 *μ*m.

The 3D microfluidic coil spring diode in this study comprised a helical coil spring connected to a cylindrical sealing disc and several pathways for fluid flow, including a bottom orifice, an intermediary structure with a center orifice and eight radially arrayed through-holes, and a top orifice (Fig. 4a). Under forward flow conditions, fluid from the microchannel enters the element through the bottom orifice, bringing the sealing disc into contact with the intermediary structure as the coil spring compresses (Fig. 4a–*left*). The intermediary structure serves as a physical boundary to prevent large spring deformations (*e.g*., Supplementary Movie S3) that can result in spring/disc misalignment-based failures. Despite the sealing disc blocking flow through the center orifice of the intermediary structure, fluid is able to continue flowing through the radially arrayed through-holes and out the top orifice (Fig. 4a–*left*). When the flow polarity is reversed, however, the coil spring extends to allow the cylindrical disc to seal atop the bottom orifice, thereby physically obstructing fluid flow through the microfluidic diode element (Fig. 4a–*right*).

To provide insight into the theoretical flow dynamics of the designed 3D microfluidic coil spring diode, we performed finite element analysis (FEA) fluid-structure interaction (FSI) simulations of the element (Fig. 4b; Supplementary Movie S4). The simulation results revealed fundamental differences associated with each directional flow polarity (*see* Supplementary Text; Supplementary Fig. S3). In particular, as the input pressure was incrementally increased in the forward flow direction, the magnitude of the flow rate also increased, with the caveat that these effects were non-linear due to the varying resistive effects corresponding to the decreasing distance between the sealing disc and the intermediary structure. After the sealing disc was immobilized, the flow rate increased linearly with increasing pressure. For the reverse flow case, the fluid flow rates through the element were similar to those of the forward flow case for lower pressures. As the reverse pressure continued to increase, however, the magnitude of fluid flow decreased until the sealing disc fully descended onto the bottom orifice and the fluid flow ceased (Fig. 4b; Supplementary Movie S4; Supplementary Fig. S3).

**Figure 5:**
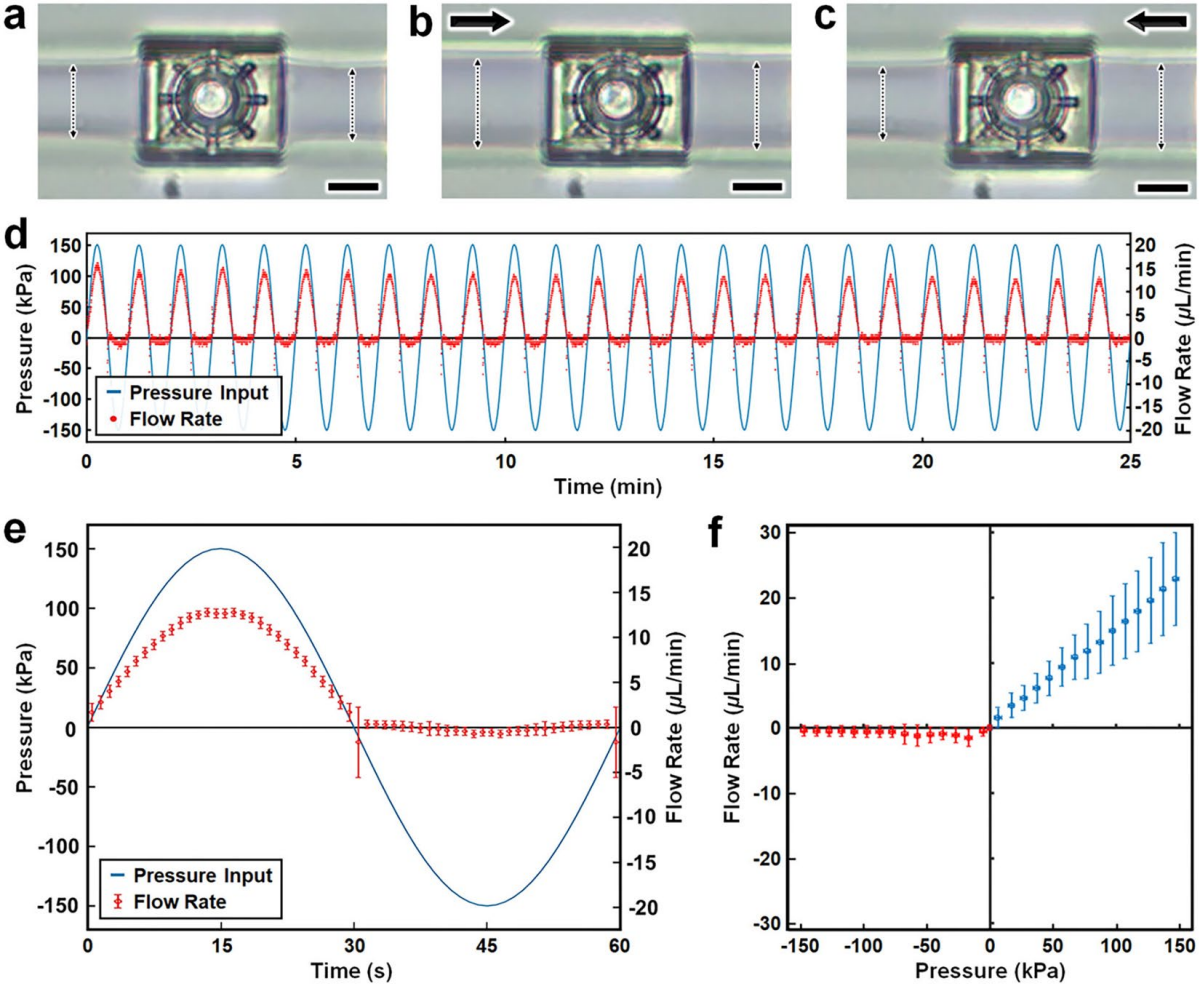
Experimental results for the *is*DLW-printed 3D microfluidic coil spring diode. (**a**–**c**) Micrographs of PDMS channel expansion adjacent to the microfluidic diode corresponding to applied pressures of: (**a**) 0 kPa (control), (**b**) 150 kPa in the forward direction, and (**c**) 150 kPa in the reverse direction (*see also* Supplementary Movie S6). Arrows denote the direction of applied pressure; dotted double arrows mark the PDMS microchannel width; Scale bars = 10 *μ*m. (**d,e**) Half-wave fluidic rectification results for (**d**) 25 minutes of testing, and (**e**) a single 60 second period averaged for three distinct tests. (**f**) Quantified results for directional fluid flow *versus* pressure. All error bars denote standard deviation; negative values for pressures and flow rates denote positive pressures and flow rates in the reverse direction.

We applied the aforementioned *is*DLW protocols to fabricate the 3D microfluidic diodes within sol-gel-coated PDMS-on-glass microchannels with semi-ovular cross-sectional profiles and approximately 25 *μ*m height. Sequential CAM simulations and fabrication results are presented in Fig. 4c (*see also* Supplementary Movie S5). Preliminary flow rectification experiments revealed the effects on the expansion of the PDMS microchannels adjacent to the microfluidic diode (Fig. 5a–c; Supplementary Movie S6). Due to the highly compliant nature of PDMS, increasing the input pressure resulted in an observable enlargement of the microchannel walls. For example, under an input pressure of 150 kPa in the forward flow direction, the microchannel walls on both sides of the diode showed significant expansion compared to their non-pressurized state (Fig. 5a,b). In contrast, for an input pressure of 150 kPa in the reverse direction, expansion of the microchannel walls was only observed upstream of the microfluidic diode, with no apparent downstream wall deformation (Fig. 5c; Supplementary Movie S6).

The ability for a microfluidic diode to serve as a half-wave fluidic rectifier is a critical metric of element functionality^54^. To test this capability, we introduced sinusoidal input pressures, which entailed repeatedly cycling the pressure from 150 kPa applied in the forward direction to 150 kPa applied in the reverse direction over a period of 60 s (Fig. 5d,e). The results revealed significant forward bias of the flow polarity, with the flow rate behavior closely matching the pressure changes for the forward direction, yet restricted flow rates despite increasing pressures in the reverse direction. For each change in the flow polarity from forward to reverse pressure as well as reverse to forward pressure, we observed initial spikes in the flow rate that quickly dissipated within approximately 2 seconds (Fig. 5e). We also found that the amplitude of the forward flow peaks appeared to wane slightly with time (Fig. 5d). An additional figure of merit for microfluidic diodes is the non-dimensional *Diodicity* (*Di*), which is calculated as:

1 $$Di=\frac{{R}_{Reverse}}{{R}_{Forward}}$$

where *R* is the hydrodynamic resistance associated with forward and reverse flow^54,55^. To characterize the *Di* performance of the 3D microfluidic coil spring diode, we further quantified the polarity-based flow behavior through non-oscillating experiments. These experiments provided insight into the steady-state flow rates associated with distinct forward and reverse pressures (Fig. 5f). The forward flow dynamics exhibited linear increases in the magnitude of fluid flow up to 22.8 ± 7.2 *μ*L/min at 147.4 ± 2.5 kPa. This was in stark contrast to the reverse flow results, which revealed significant obstruction of fluid flow, with different flow behaviors at lower and higher pressures. Specifically, for pressures applied in the reverse direction of up to 67.4 ± 2.4 kPa, the average flow rate appeared to fluctuate between 0.61 ± 0.71 and 1.6 ± 1.3 *μ*L/min. For reverse pressures from 77.3 ± 2.6 kPa to 147.4 ± 2.4 kPa, however, the average flow rate was consistently maintained below 0.64 ± 0.88 *μ*L/min (Fig. 5f). These results correspond to an overall trend of improved *Di* performance at higher pressures, with a maximum *Di* of approximately 45.8 at the largest pressure magnitudes tested approaching 150 kPa.

## Discussion

The sol-gel-based *is*DLW strategy and results presented in this work provide a fundamental foundation for emerging classes of microfluidic technologies that benefit from the 3D architectural control and submicron-scale resolution inherent to DLW, while bypassing the impediments that stem from facilitating the micro-to-macro interfaces that are critical to system utility. Data from this systematic characterization of the role of microchannel geometry in the sealing efficacy of *is*DLW-printed microstructures offers new means to inform the design of future *is*DLW-based microfluidic systems. Experiments with microfluidic barrier walls revealed a correlation between larger microchannel height and reduced sealing integrity (Fig. 3). One caveat, however, is that the total force acting on the barriers is linearly related to the surface area of the structure itself (*see* Supplementary Text; Supplementary Fig. S4). Thus, for an identical pressure, barriers printed in taller microchannels sustained substantially larger forces. These disparities in applied force offer a potential basis for the observed differences in sealing performance with respect to microchannel height (Fig. 3), particularly because the thickness of the barrier walls was maintained at 10 *μ*m regardless of height. If these force disparities account for the observed reductions in sealing integrity with increasing height (rather than alternative mechanisms, such as diminished adhesion at taller heights), then such sealing failures could be mitigated by increasing the attachment area between *is*DLW-printed structures and the luminal surface of the sol-gel-coated microchannel.

The burst-pressure experiments for the barrier structures also revealed that the shape of the microchannel cross-section serves as a key determinant of microfluidic sealing performance (Fig. 3). In general, the semi-circular, semi-ovular, and triangular channel profiles outperformed the profiles designed to mimic conventional microfabrication-based microchannels–results that may be due to a number of potential factors. Similar to changes in height, differences in the shape, and in turn, surface area of the barrier structure can lead to disparities in the applied force for a given pressure. Theoretical simulations of the barrier walls revealed that the semi-circular profile resulted in the largest sustained forces and stresses (*see* Supplementary Text; Supplementary Fig. S4). Thus, if shape-based differences in applied force influence the sealing functions of barriers of the same height, then the semi-circular profile should exhibit the worst performance of all of the profiles examined. Experiments revealed that this was not the case, with the semi-circular geometry yielding superior performance compared to many of the other profiles for heights of 10, 25, and 50 *μ*m (Fig. 3). These results suggest that force disparities cannot account for the shape-based differences in sealing integrity, which were likely caused by alternative mechanisms.

One of the trends elucidated during burst-pressure testing was that increasing the outward tapering of the microchannel sidewalls generally corresponded to improvements in sealing performance (Fig. 3). In addition, several cases for the conventional microfabrication-inspired profiles (Fig. 3a–c) exhibited leakage flow from the onset of pressure testing, which implies the absence of luminal adhesion prior to experiment initiation. A potential basis for these results stems from fabrication issues that led to a lack of sealing continuity between printed barrier structures and the channel walls (*e.g*., Supplementary Fig. S5). In particular, printing in certain locations within the microchannel (*e.g*., the top corners of the rectangular, inward-tapered, and outward-tapered channels) can lead to unintended focusing deviation caused by interactions between the laser path and the lower portions of the PDMS through which the laser passes. Such disruptions of the laser path can inhibit photopolymerization initiation, thereby preventing curing of the photomaterial in specific locations–a phenomenon termed “shadowing”^36^. Additionally, these effects would be exacerbated in cases with taller channel heights, which include a longer distance in which disruptive laser-PDMS interactions can occur. The observed differences in sealing behaviors associated with the distinct tapering of the various microchannel profiles are consistent with those predicted by shadowing phenomena (Fig. 3). One note is that it may be possible to mitigate such shadowing effects by dynamically adjusting the laser power and/or scanning speed during the *is*DLW fabrication process to promote photoinitiation in shadowed regions; however, elucidating the optimal parameters to achieve successful prints may be experimentally challenging due to the difficulties in optically characterizing nanostructured features within enclosed microchannels. In contrast, we found that the use of semi-circular, semi-ovular, and triangular channel profiles bypassed the need for such experimental optimizations. Thus, due to the critical requirement for sufficient side-wall tapering in order to avoid shadowing failure modes, the results suggest that conventional soft lithography protocols are ill-suited for *is*DLW.

Although the semi-circular microchannel yielded effective barrier sealing for channel heights up to 50 *μ*m, the 100-*μ*m-tall profile exhibited fluid leakage from the onset of burst-pressure testing (Fig. 3d). The key difference between the 100 *μ*m case and all of the other barrier structures was that the manufacturing restrictions of the DLW printer (build area ≈125 × 125 *μ*m^2^) required that the 200-*μ*m-wide barrier wall be fabricated in two separate parts (Supplementary Movie S2). The results suggest that for this barrier structure design, effective joining of the two parts did not occur, leading to immediate leakage during testing (Fig. 3d–*yellow*). Thus, the observed leakage for the 100-*μ*m-tall profile was likely a product of the multi-step fabrication process rather than adhesion issues between the barrier structure and the sol-gel-coated PDMS.

The 3D microfluidic coil spring diode in this work represents, to the best of our knowledge, the smallest mechano-fluidic diode^54–58^ and the smallest 3D printed mechano-fluidic circuit element^51–54^ reported in the literature. One caveat to this scale, however, is that fluidic operation is more susceptible to debris in the microchannel. Although we implemented filters to mitigate the effects of internal debris, the half-wave fluidic rectification results suggest that debris accumulation in the filters contributed to slight reductions in the maximum flow rates at the onset of fluidic testing (Fig. 5d). Specifically, flow rectification experiments for the microfluidic diode over 100 cycles revealed that such reductions were limited to the first 20 cycles as the overall flow behavior remained relatively constant thereafter (Supplementary Fig. S6). The experimental results for half-wave flow rectification also revealed brief spikes in the flow rate corresponding to each reversal in the flow polarity (Fig. 5d,e). Two main factors could account for such behavior: (*i*) physical resistance to fluidic sealing due to a large coil spring stiffness, and (*ii*) fluid volume discharge due to the hydraulic capacitance of the PDMS channels. Based on the coil spring geometry and material properties, the coil spring stiffness is estimated to be on the order of 1 nN/*μ*m–a stiffness that resulted in significant deformation of the spring at low pressures (Supplementary Movie S3). Thus, it is more likely that the ability for the PDMS microchannels to operate as hydraulic capacitors^57,58^ led to the aforementioned flow rate spikes. Under an applied pressure, the PDMS microchannels expand to store fluid volume; however, once the pressure is no longer applied, the PDMS microchannels contract to their initial state, releasing the previously stored fluid volume. Experimentation revealed expansion-contraction behaviors of the PDMS microchannels that are consistent with hydraulic capacitor functionalities (Fig. 5a–c; Supplementary Movie S6). Nonetheless, the overall fluidic rectification functionalities of the *is*DLW-printed 3D microfluidic diode are unprecedented at this scale (Fig. 5d–f). Due to emerging applications in fields such as soft robotics^59,60^ and biofluidic processing^51–53^ that rely on the scaling of microfluidic circuitry, the 3D microfluidic diode in this study could serve as an important baseline for a new generation of microfluidic circuit elements that are constructed by means of *is*DLW.

Similar to numerous additive manufacturing approaches, 3D computer models of microchannels and *is*DLW-printable structures can be electronically disseminated for rapid adoption and reproduction of state-of-the-art advancements. Because the presented sol-gel-based *is*DLW strategy is founded on DLW process steps, conventional microfabrication facilities and equipment are not required for replicating and/or applying the methodologies described in this work. As a result, critical barriers that have historically limited the utilization of submicron-scale methods of fluidic device manufacturing to those in traditional nanoengineering fields can by bypassed, opening new pathways for investigators from diverse academic disciplines to leverage the benefits afforded by these scales for wide-ranging chemical, mechanical, and biological applications.

## Methods

### Microfluidic device fabrication

For fabrication of the mold master, 3D models of the channel designs were created using the computer-aided design (CAD) software, SolidWorks (Dassault Systemes, France). The CAD models were then imported into the CAM software, DeScribe (Nanoscribe GmbH, Germany), and converted to writing-path code. Si substrates (25 mm × 25 mm) were successively rinsed with acetone and isopropyl alcohol (IPA), dried with inert N_2_ gas, and then baked at 100 °C for 15 minutes. The Nanoscribe Photonic Professional GT (Nanoscribe) was used with a 25 × objective lens in DiLL mode to print the master molds comprised of the negative-tone photoresist, IP-S (Nanoscribe), onto the Si substrates (Fig. 1a). The channel molds were fabricated with layer heights of 1 *μ*m and hatching distances of 500 nm (Supplemental Fig. S7). For DLW, the laser power and scan speed were set to 45 mW and 100 mm/s, respectively. The writing times for the negative master molds were 6, 10.5, 11, and 12.5 minutes corresponding to channel heights of 10, 25, 50, and 100 *μ*m, respectively. Following the DLW process, substrates were developed first in a bath of propylene glycol monomethyl ether acetate (PGMEA) for 20 minutes, and then in IPA for 2 minutes to remove any remaining uncured photoresist (Fig. 1b). A 10:1 mixture of PDMS (Sylgard 184, Dow Corning, Corning, NY) was then poured over the master molds and placed on a hot plate set at 60 °C for 3 hours (Fig. 1c). Cured PDMS was then peeled from the molds and punched with 0.75 mm at inlet and outlet locations. The PDMS was rinsed with IPA, and then O_2_ plasma bonded to 30 mm circular borosilicate glass substrates (#1.5, Bioptechs Inc., Butler, PA) (Fig. 1d).

### Sol-gel coating

The PDMS-on-glass microfluidic devices were chemically coated using an acid-catalyzed sol-gel reaction presented by Beal *et al*.^47^ (Fig. 1e). First, a solution of 33% APTES in ethanol was perfused into the channels for 5 minutes and cleared with air pressure. Then, a solution of 33% Acetic Acid was perfused into the channel for 3 minutes, catalyzing APTES onto the surface of the PDMS channel. Lastly, the Acetic Acid was cleared from the channels with air pressure, and the device was placed on a hot plate set at 100 °C for 5 minutes.

### Microstructure *is*DLW fabrication

For fabrication of both the barrier wall structures and the 3D microfluidic coil spring diode, 3D models of the designs were created using SolidWorks (Dassault Systemes) and then imported into DeScribe (Nanoscribe) for writing-path code generation. The negative-tone photoresist, IP-L 780 (Nanoscribe), was vacuum-loaded into the sol-gel-coated PDMS-on-glass microchannels (Fig. 1f). The Nanoscribe Photonic Professional GT (Nanoscribe) was used with a 63× objective lens in oil-immersion mode to print the structures inside of the microchannels (Fig. 1g). Briefly, this printing strategy involves placing a droplet of immersion oil between the objective lens and the bottom of the glass substrate to maintain the focal path of the laser. All microstructures were printed in a “ceiling-to-floor”, point-by-point, layer-by-layer process. Following *is*DLW completion, the microfluidic devices were placed in a bath of PGMEA for approximately 4 hours. Therafter, the Fluigent Microfluidic Control System (MFCS) (Fluigent, France) was used to perfuse PGMEA through the channels for 5 minutes, and then IPA for 1 minute at pressures of <10 kPa.

### Theoretical simulations

All FEA simulations were performed using the commercial software, COMSOL Multiphysics v.5.3a (COMSOL Inc., Sweden). Initially, 3D models of the components of interest (*e.g*., barrier walls, microfluidic diodes) were created using SolidWorks (Dassault Systemes). The 3D CAD models were then imported into the COMSOL Multiphysics software. For the fluidic barrier walls, static solid mechanics simulations were performed while fixing the lateral surfaces of the barrier (which would be connected to the luminal surface of the microchannel) and increasing the applied pressure on one side of the barrier wall from 0 to 75 kPa. Von Mises stress profiles were outputted for analysis. For the microfluidic diode, FSI simulations were performed with the stokes flow physical model and quasi-static structural transient behavior. The structure material (IP-L 780) was modeled with material properties E = 1.75 GPa and *ν* = 0.49^61^. The input pressure conditions were designed to ensure simulation termination upon mesh intersection (*e.g*., the surface of the sealing disc interacting with either the bottom orifice or the intermediary structure the source output channel) in order to avoid topological changes. IPA (*ρ* = 0.783 kg/m^3^; *η* = 2.04 mPa $\cdot$ s) was modeled as the input fluid.

### Experimental setup and analysis

In all experimental tests, MAESFLO software (Fluigent), which operates the MFCS and Flow Rate Platform, was utilized to regulate input pressures and record concurrent pressure and flow rate data during IPA perfusion through microfluidic channels at room temperature (20–25 °C). Microchannels were connected to the MFCS through fluorinated ethylene propylene tubing (Cole-Parmer, Vernon Hills, IL) and 20 ga. stainless steel catheter couplers (Instech, Plymouth Meeting, PA). Experiments were conducted on at least three separately fabricated components, and all results were compiled, processed, and plotted using a binning MATLAB script. For burst-pressure testing of the barrier walls, an MFCS pressure source was connected to the device on one side of the barrier, and outlet tubing was connected to the device on the other side of the barrier, while the remaining two ports were sealed with 20 ga. stainless steel plugs (Instech). Flow units were connected in series to the inlet and outlet tubing to record the magnitude of fluid flow on each side of the barrier. A script written in the Fluigent software was used to increase the pressure input in a stepwise manner from 0 kPa to 75 kPa with increments of 2.5 kPa and a 10 s settling time.

For experimental testing of the microfluidic diode, all input parameters were controlled using scripts written in the Fluigent software, while flow rate measurements were collected from two flow units (connected to each end of the straight channel). For the half-wave rectification tests, three microfluidic diodes were tested by introducing a sinusoidal pressure input to the microchannel, with an amplitude of 150 kPa and time period of 60 s. For the steady-state flow behavior characterization, a total of 12 tests were performed using three different diodes. The pressure was first increased from 0 to 150 kPa in forward flow configuration with a 5 kPa step size and 10 s settling time–parameters set by the operational capabilities of the MFCS platform. Similarly, the procedure was repeated in the reverse flow configuration after a 30 s settling period. The data collected from both testing procedures were processed and plotted in MATLAB.

## Electronic supplementary material

Supplementary Information

Supplementary Movie S1

Supplementary Movie S2

Supplementary Movie S3

Supplementary Movie S4

Supplementary Movie S5

Supplementary Movie S6
