# Supplementary material for: Geometric Determinants of In-Situ Direct Laser Writing
Source: Sci Rep. 2019 Jan 23;9:394. doi: 10.1038/s41598-018-36727-z (PMC6344532; doi:10.1038/s41598-018-36727-z)
Supplement: Supplementary file 1 — Supplementary Information [file 41598_2018_36727_MOESM1_ESM.pdf]

Geometric Determinants of *In-Situ* Direct Laser Writing

Andrew C. Lamont[1,2], Abdullah T. Alsharhan[1], and Ryan D. Sochol[1,2,*]

[1]Department of Mechanical Engineering
[2]Fischell Department of Bioengineering
[*]2147 Glenn L. Martin Hall, University of Maryland, College Park, MD 20740. Email: rsochol@umd.edu

Supplementary Information

Supplementary Text

Barrier wall theoretical simulation results and discussion

We performed theoretical FEA simulations to elucidate the differences in von Mises stress in response to identical pressures applied to barrier walls with varying cross-sectional profiles and heights, but identical thicknesses of 10 μm (**Supplementary Fig. S4**). The applied force on barrier wall is proportional to the area of the surface on which the pressure is applied. Accordingly, decreasing the barrier height for a given profile from 100 μm (**Supplementary Fig. S4** – *top*) to 10 μm (**Supplementary Fig. S4** – *bottom*) universally led to a dramatic reduction in internal stress. These results suggest that if the mechanical integrity of the barrier walls is a critical factor in sealing functionality, then the barriers with larger heights would fail at lower pressures. Although the experimental results revealed trends consistent with this prediction (**Fig. 3**), it is unclear if stress was the primary cause or merely contributed in part to the reductions in sealing performance for barriers with larger heights as a number of additional factors (*e.g.*, differences in structure-wall adhesion surface area, height-based shadowing effects) could also account for such disparities.

Similar to the differences in height, barrier walls that included profiles with larger surface areas resulted in increased von Mises stress propagation (**Supplementary Fig. S4** – *top*). Based on the stress distributions, if barrier wall mechanical integrity plays a significant role in sealing efficacy, then it would be expected that the semi-circular profiles (**Supplementary Fig. S4**d) would exhibit the worst performance, followed by the rectangular profile (**Supplementary Fig. S4a**), then the outward-tapered, inward-tapered, and semi-ovular profiles (**Supplementary Fig. S4b,c,e**), and lastly the triangular profile (**Supplementary Fig. S4f**). In contrast, experimental testing revealed that the semi-circular profiles—along with the semi-ovular and triangular cross-sections—typically demonstrated the strongest sealing integrities (**Fig. 3**). It is important to note that the unique two-step DLW fabrication requirement for the 100-μm-tall semi-circular barriers (**Supplementary Movie S2**) renders it inadequate for stress-based performance comparisons. Despite having identical surface areas and stress profiles (**Supplementary Fig. S4b,c**), the outward-tapered profile generally exhibited improved sealing functionalities compared to the inward-tapered profile (**Fig. 3b,c**). In combination, the disparities between the performance predicted by the simulation results and the results observed during experimental burst-pressure testing suggest that alternative mechanisms, rather than stress propagation, served as determining factors in sealing efficacy.

Microfluidic coil spring diode theoretical simulation results and discussion

To provide insight into the ideal functionalities of the microfluidic coil spring diode, we quantified the magnitude of the fluid flow with respect to input pressure for the theoretical FEA simulations (**Fig. S3**; **Fig. 4b**; **Supplementary Movie S4**). The simulation results revealed non-linearities in the flow behavior for both polarities. For pressures up to approximately 5 Pa applied in the forward direction, the flow rate appeared to increase linearly with the pressure. As the pressure continued to increase, the sealing disc approached the center orifice of the intermediary structure, which resulting in fluctuating increases in the hydraulic resistance, and in turn, impeded the flow of fluid through the element. In particular, we observed a relatively larger decrease in the rate of forward flow from approximately 9 Pa to 13 Pa, at which point the disc fully sealed to the intermediary structure (**Fig. S3** – *red line*). For pressures applied above 13 Pa, the flow rate appeared to linearly increase with increasing pressure; however, the rate at which the flow increased with pressure—a function of the hydraulic resistance through the element—was slightly smaller than that observed from 0 Pa to 5 Pa. Although this behavior indicates that the hydraulic resistance through the element is slightly larger after the disc seals to the intermediary structure, the effect was limited to a nominal reduction in the overall magnitude of forward flow. For the reverse flow case, from 0 Pa to approximately -5 Pa, the flow behavior was essentially indistinguishable from that of the forward flow case for the same range of pressures. From -5 Pa to -10 Pa, however, the rate at which the reverse flow increased with increasing reverse pressure began to decrease, with a peak reverse flow at -10$\pm$1 Pa. Thereafter, the magnitude of reverse flow through the microfluidic element declined substantially as the sealing disc approached the bottom orifice, with full sealing interactions by -18 Pa (**Fig. S3**).

Supplementary Figures

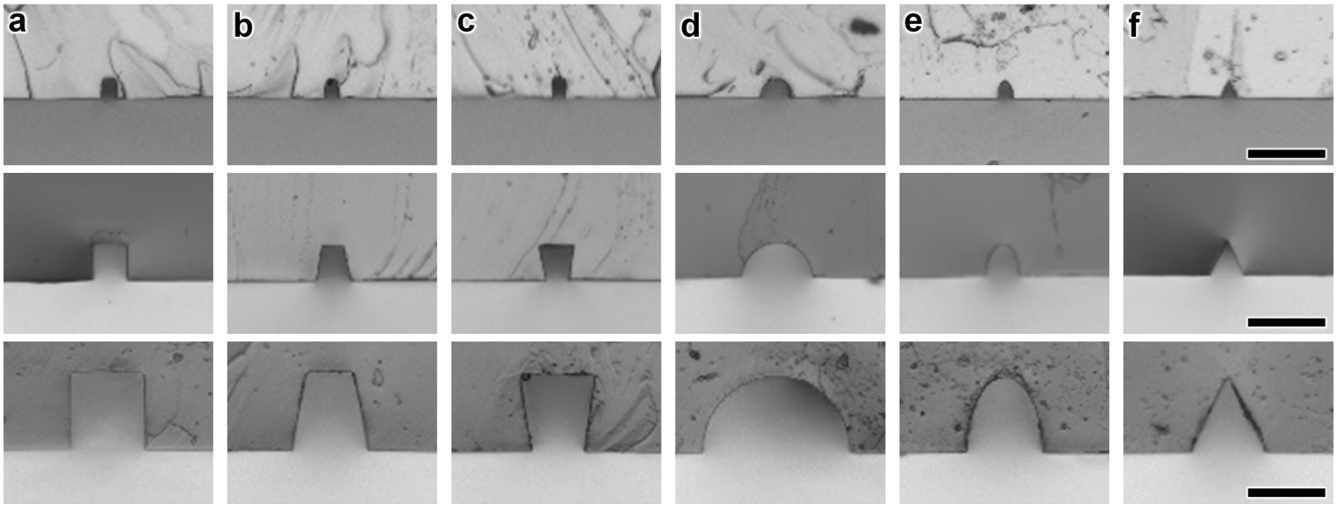

Figure S1. Micrographs of replicated PDMS profiles for heights of 10 μm (*Top*), 25 μm (*Middle*), and 50 μm (*Bottom*) corresponding to distinct microchannel cross-sectional profiles: (**a**) rectangular (deep reactive-ion etching (DRIE) mimetic), (**b**) outward-tapered (positive-tone photoresist mimetic), (**c**) inward-tapered (negative-tone photoresist mimetic), (**d**) semi-circular, (**e**) semi-ovular, and (**f**) triangular. Scale bars = 50 μm

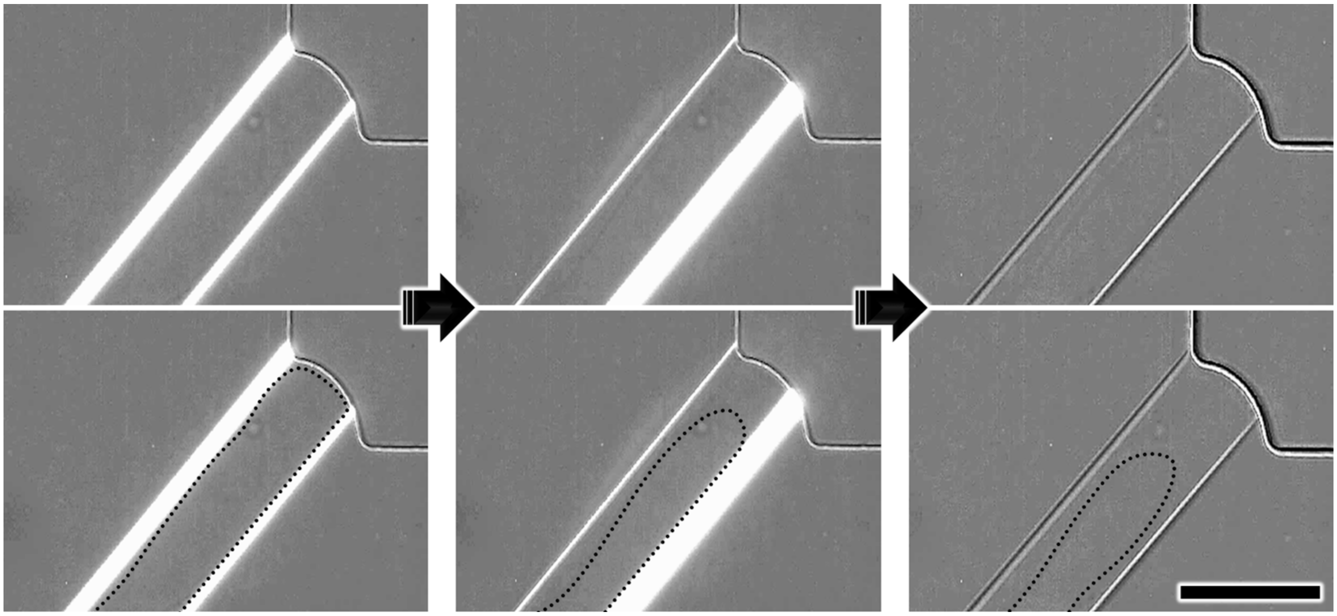

Figure S2. Sequential micrographs of barrier wall detachment from the top surface of an uncoated PDMS microchannel during *is*DLW fabrication without (*Top*) and with (*Bottom*) dotted lines (for visualization purposes) marking the area of the barrier wall that remains attached to the top surface of the PDMS channel. Scale bar = 50 μm

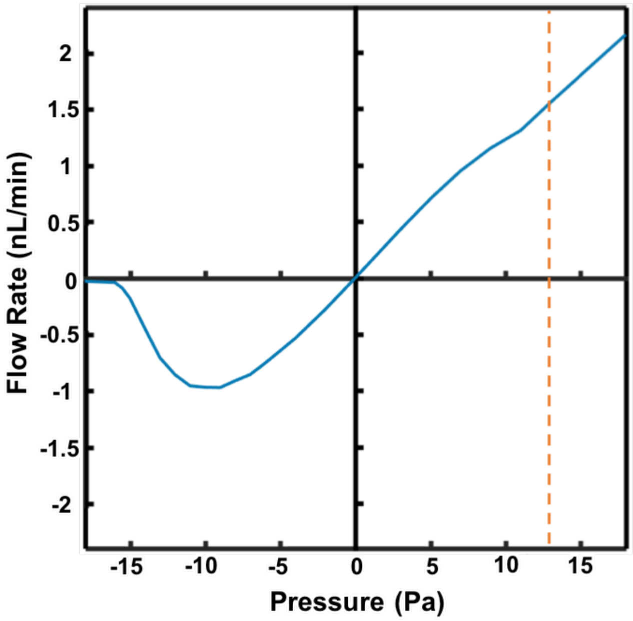

Figure S3. Quantified simulation results for the polarity-based flow dynamics of the 3D microfluidic coil spring diode. The dashed red line marks the pressure at which the sealing disc fully contacted the intermediary structure, physically blocking flow through the center orifice.

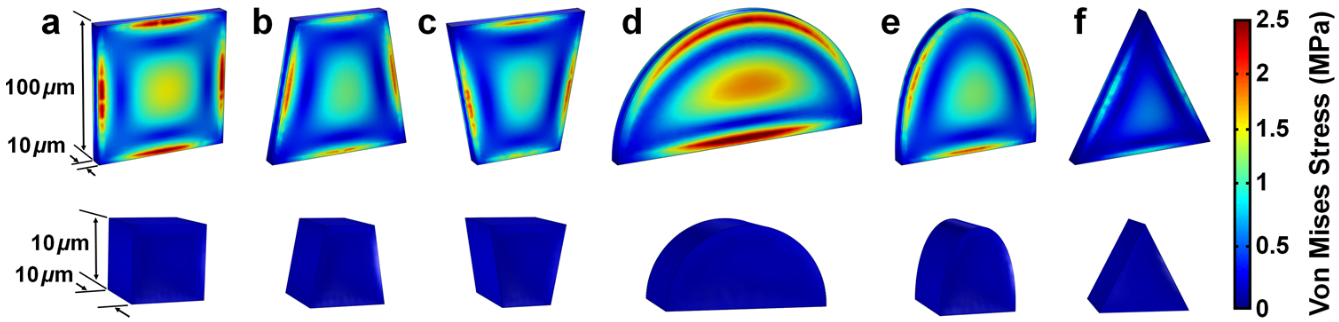

Figure S4. Theoretical simulation results for von Mises stress distributions for barrier wall structures with heights of 100 μm (*Top*) and 10 μm (*Bottom*) corresponding to distinct microchannel cross-sectional profiles: (**a**) rectangular (deep reactive-ion etching (DRIE) mimetic), (**b**) outward-tapered (positive-tone photoresist mimetic), (**c**) inward-tapered (negative-tone photoresist mimetic), (**d**) semi-circular, (**e**) semi-ovular, and (**f**) triangular.

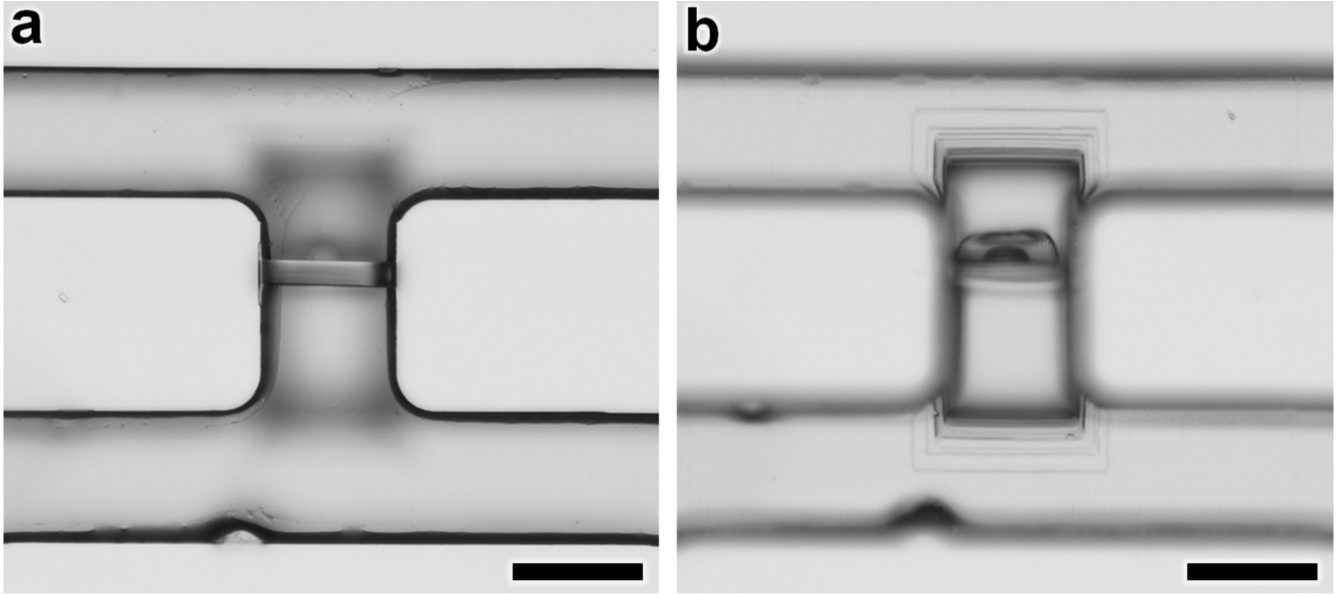

Figure S5. Fabrication results for a "floor-to-ceiling" *is*DLW process for a 10-μm-thick barrier wall structure printed within a 50-μm-tall sol-gel-coated PDMS microchannel with a square cross-sectional profile. (**a**) Micrograph focused at the bottom glass surface of the microchannel. (**b**) Micrograph focused at the top surface of the barrier wall. Scale bars = 50 μm

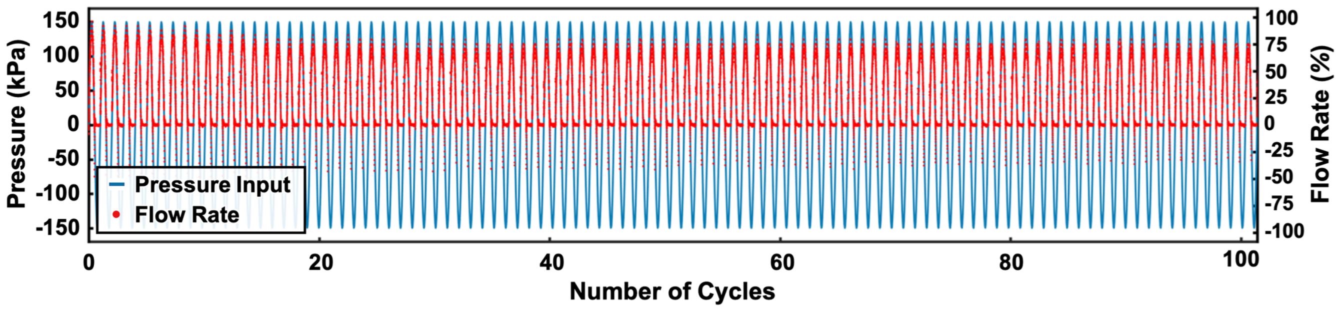

Figure S6. Experimental half-wave fluidic rectification results for the *is*DLW-printed 3D microfluidic coil spring diode for 100 cycles of forward and reverse flow polarity.

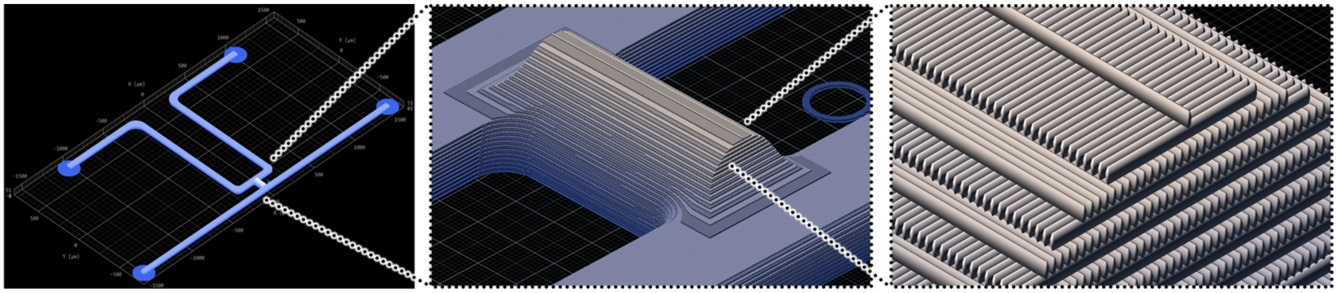

Figure S7. CAM simulation of the writing path of the scanning pulsed laser for a microchannel master mold with a 25-μm-tall semi-ovular channel profile and four inlet/outlet port locations.

Supplementary Movie Captions

Movie S1. CAM simulation (*Left*) and corresponding *is*DLW fabrication results (*Right*) for the *is*DLW-based printing of a microfluidic barrier wall structure (10 μm in thickness) within a sol-gel-coated PDMS microchannel with a triangular cross-sectional profile and a height of 50 μm. Video speed = 4×; Scale bar = 50 μm

Movie S2. Fabrication of a 100-μm-tall barrier wall in a sol-gel-coated PDMS-on-glass microchannel with a semi-circular cross-sectional profile. Video speed = 10×; Scale bar = 30 μm

Movie S3. Deflection behavior of an *is*DLW-printed helical coil spring in response to varying pressure input. Video speed = 10×; Scale bar = 40 μm

Movie S4. Theoretical simulation results for the microfluidic coil spring diode under (**a**) forward flow, and (**b**) reverse flow conditions. Structure color represents displacement in μm; Arrows represent fluid velocity field vectors

Movie S5. *Is*DLW fabrication results for printing a 3D microfluidic coil spring diode within a sol-gel-coated PDMS-on-glass microchannel with a semi-ovular cross-sectional profile and height of ∼25 μm. Video speed = 6×; Scale bar = 10 μm

Movie S6. Deformation behavior of the PDMS channel during the transition from pressures of 0 kPa to 150 kPa applied in the (**a**) forward flow direction (*rightward*), and (**b**) reverse flow direction (*leftward*). Scale bar = 10 μm
